# High Level Secretion of Laccase (LccH) from a Newly Isolated White-Rot Basidiomycete, *Hexagonia hirta* MSF2

**DOI:** 10.3389/fmicb.2016.00707

**Published:** 2016-05-18

**Authors:** Sujatha Kandasamy, Iniya K. Muniraj, Namitha Purushothaman, Ashika Sekar, D. J. S. Sharmila, Ramasamy Kumarasamy, Sivakumar Uthandi

**Affiliations:** ^1^Department of Agricultural Microbiology, Tamil Nadu Agricultural UniversityCoimbatore, India; ^2^Department of Nanoscience and Technology, Tamil Nadu Agricultural UniversityCoimbatore, India

**Keywords:** Laccase, *Hexagonia hirta*, high level secretion, physiological regulation, bio delignification

## Abstract

Newer and novel laccases attract considerable attention due to its promising and valuable multiple applications in biotech industry. This present investigation documents, for the first time, on high level extracellular secretion of laccase (LccH) in newly isolated wood-degrading basidiomycete *Hexagonia hirta* MSF2. LccH was optimally active at 40°C in citrate phosphate buffer with a pH of 3.4. Optimized Cu^2+^ in glucose yeast extract (GY) medium enhanced the LccH production by *H. hirta* to 1944.44 U.ml^-1^. A further increment in LccH activity of 5671.30 U.ml^-1^ was achieved by the addition of a phenolic inducer, 2,5 Xylidine. Zymogram and sodium dodecyl sulfate–polyacrylamide gel electrophoresis (SDS–PAGE) analysis of LccH revealed that LccH is a monomer with a molecular mass of 66 kDa. MALDI-TOF-MS based peptide mass fingerprinting and comparative modeling of the amino acid sequence of LccH showed that it was closer to *Trametes* sp. AH28-2 (PDB: 3KW7) with 48% identity, 95% coverage, 0.011 alignment score and RMSD of 0.497Å. Crude LccH delignified lignocellulosic biomass such as wood and corncob, to a level of 28.6 and 16.5%, respectively. Such high level secretion, thermal and solvent stability of LccH make *H. hirta* a potential candidate not only for LccH production and biodelignification but also generation of lignin derived aromatic feed stock chemicals for industrial and environmental applications.

## Introduction

Laccases (benzenediol: oxygen oxidoreductase EC 1.10.3.2) are glycosylated blue oxidases, that oxidizes wide range of substrates including aromatic amines, diamines, diphenols, polyphenols, and highly recalcitrant environmental pollutants with simultaneous reduction of molecular oxygen to water (Baldrian, 2006; You et al., 2013). Although laccases from plants, fungi, prokaryotes, arthropods (Wang et al., 2000), and archaea (Uthandi et al., 2010) are widely described, white-rot fungi are the most predominant group that are known for high level secretion of laccases (Wu et al., 2010; Yang et al., 2013b). Due to their capability to oxidize a wide range of compounds, laccases in the last decade have been considered as potential enzyme for industrial applications such as cellulose pulping, dye decolourization, detoxification of phenolics from industrial effluents, biosensor applications, food and beverage industries, cosmetic industry and nano biotechnology, and bioremediation of soils (Soden and Dobson, 2001; Morozova et al., 2007; Shraddha et al., 2011; Diwaniyan et al., 2012; Virk et al., 2012; Ba et al., 2013; Christopher et al., 2014). Therefore, owing to their broad range of applications laccases are considered as one of the greenest enzymes in the 21st century (Mate et al., 2013) and their use in industry has been growing rapidly over the past decade.

To meet the increasing demand, production of large amounts of enzyme at a lower cost is required for commercial viability. Most studies related to laccase production for industrial applications are targeted on recombinant fungal strains (Catalano et al., 2011; Galai et al., 2011). Although, recombinant strains are capable of overproducing laccase, cloning and expression of laccase in a recombinant host is overwhelmed by problems, such as different codon usage, missing chaperone and post translational modifications. In addition, recombinant laccases tends to have a lower redox potential which consequently reduces their specific activity compared to those from wild strain (Alves et al., 2004).

Hence, search for new laccase producing native isolates becomes imperative on the face of requirement for higher titres, broad substrate specificity and tolerance to various conditions such as, high temperatures, pH and organic solvents. In addition, strategies for their overproduction at lower cost are also needed. Several researchers are constantly engaged in identifying new organisms for laccase production. For instance, *Trametes trogii* TEM H2 a newly isolated fungi from air samples have been shown to produce laccase in high titres (Kocyigit et al., 2012). Similarly, laccase from a newly isolated *Shiraia sp*. SUPER-H168 was reported to have a laccase yield of 101 U.ml^-1^ (Yang et al., 2013a). Laccase from a new psychrotolerant fungus *Cladosporium tenuissimum* isolated form cold deserts of Indian Himalayas was reported to posses enhanced cold stability (Dhakar and Pandey, 2015). In the present study we report for the first time, the high level secretion of laccase (LccH) from a newly isolated basidiomycetous fungus *Hexagonia hirta* MSF2 and its potential application in delignification of lignocellulosic biomass.

## Materials and Methods

### Materials

Biochemicals were from Sigma–Aldrich India (Bengaluru). Other organic, inorganic analytical grade chemicals, and agarose were from HI-Media Laboratories Pvt. Ltd (Mumbai). Molecular biological chemicals were from New England Biolabs (Gurgaon, India) and Takara India (New Delhi). Desalted oligonucleotides were synthesized from Eurofins Analytical Services India Pvt. Ltd (Bengaluru).

### Isolation and Screening of Laccase Producing Isolate MSF2

Samples were collected from various ecological niches including, decaying wood, leaf compost, and soils from the forest plantations of Forest College and Research Institute, Mettupalayam (11.2891^o^N,76.9410^o^E) Tamil Nadu, India, for isolation of laccase-producing microorganisms. The samples were serially diluted and spread onto Potato Dextrose Agar plates (PDA) (pH 6.5) containing 0.04% guaiacol and incubated at 37°C, for 3–5 days (Kiiskinen et al., 2004). Positive colonies oxidizing guaiacol were selected. Among several fungi screened for laccase production isolate MSF2 showed higher oxidation and was selected for further experiments.

### Identification of Laccase Producing Isolate MSF2

The total genomic DNA extracted from the isolate of MSF2 using a modified phenol–chloroform extraction method was served as a template for 18S rRNA gene amplification using universal 18S rRNA pairs (NS1 {(5′-GTAGTCATATGCTTGTCTC-3′) and NS2 (5′-GGCTGCTGGCACCAGACTTGC-3′)} and ITS {(ITS 1 (5′-CTTGGTCATTTAGAGGAAGTAA-3′) and ITS 4 (5′-TCCTCCGCTTATTGATATGC-3′)} regions (O’Brien et al., 2005). Amplification was done in T100 Gradient PCR (BioRad, USA) with a total volume of 30 μl containing 1x PCR buffer with 50 mM MgCl2 10 mM of each dNTP, 10 picomol of primer, 1 U of Taq DNA polymerase (0.25 μl) and 20 ηg of genomic DNA. The thermal cycler was performed as follows: an initial hot start incubation (5 min at 94°C) followed by 34 cycles of denaturation at 94°C for 30 s, annealing at 55°C for 30 s and extension at 72°C for 1 min, followed by a final extension at 72°C for 15 min. The amplified PCR product of 555 and 625 bp for 18S rRNA and ITS, respectively, were visualized by electrophoresis in 0.8% agarose gel and documented using Biorad Gel doc XR+ system (Hercules, CA, USA). The amplicons were sequenced commercially by fluorescent dye terminators method (ABI Prism^TM^ Bigdye^TM^ Terminator cycle sequencing ready reaction kit v.3.1) and the products were purified by Millipore-montage dye removal kit. Then, the products were run in an ABI3730XL capillary DNA sequencer (50 cm capillary). The sequences of both 18S rRNA and ITS regions of isolate MSF2 were annotated and analyzed on BLAST in NCBI^1^. Phylogenetic analysis and construction of an unrooted tree were performed using the software MEGA version 3.0. The 18S rRNA sequence was submitted to NCBI (GenBank accession KT956905).

### Laccase Assay and Biochemical Analysis

Laccase production by the isolate MSF2 was monitored in PDB supplemented with 1 mM CuSO_4_ by inoculating three, five mm- mycelial plugs of MSF2 onto 250 ml Erlenmeyer flasks – containing 50 ml media and incubated at 30°C under static conditions for 10 days. Cell free supernatant was obtained by harvesting and centrifugation (10,000 × *g*, 10 min) of culture broth at 4°C. Laccase activity was determined by ABTS-oxidation at 420 nm (€ max = 3.6 × 10^4^ M^-1^cm^-1^) using multimode microplate reader (Biotek Instruments, USA). The reaction mixture contained appropriately diluted enzyme mixed with 3 mM ABTS in acetate buffer (50 mM, pH 4.5). One unit (U) of laccase activity was defined as the amount of enzyme that oxidizes 1 μmol ABTS min^-1^.ml^-1^ of the reaction mixture, under assay conditions (Sun et al., 2012).

Protein concentration in the supernatant was determined using Bradford’s reagent (Bio-Rad, Berkeley, CA, USA) by following the manufacturer’s instructions, with Bovine Serum Albumin (BSA) as the standard (Bradford, 1976). Reducing sugars in the spent medium was estimated using 3,5-dinitrosalicylic acid (DNS) reagent (Miller, 1959). Changes in pH of the medium were monitored using a pH electrode (ILECO, India). Total biomass was estimated by filtering the culture medium through filter paper No.42 (Whatman, Oxon, UK). The biomass retained was washed with distilled water and dried at 105°C to a constant weight.

### Optimization of pH, Buffers, and Temperature

The laccase from the isolate MSF2 was named as LccH and its pH optima was investigated over a pH range of 1–10 using 100 mM Britton–Robinson buffer. To find out suitable buffer for LccH, activities were evaluated in four different buffers such as 50 mM glycine-HCl buffer (pH 3.4), 50 mM sodium citrate buffer (pH 3.6), 50 mM sodium acetate buffer (pH 3.6), universal buffer (pH 3.5), and 50 mM citrate phosphate buffer (pH 3.4). The effect of temperature on the LccH activity was determined by incubating the enzyme under optimum pH at a temperature range of 30 and 50°C, under optimal pH and buffer conditions.

### Optimization of CuSO_4_ Concentrations

To find out the role of CuSO_4_ and optimal concentration required for LccH activity, CuSO_4_ was added to GY medium at concentrations ranging from 50 to 1000 μM on the 3rd day of MSF2 inoculation and incubated under the conditions previously described.

### LccH Production under Different Media

LccH production was tested in five media, *viz*., PDB (Chen et al., 2012); GY (Tlecuitl-Beristain et al., 2008); SYE (Revankar and Lele, 2006); GKP (Hou et al., 2004), and GP (Galhaup et al., 2002) with an initial pH of 6.0. Culture inoculation, incubation and LccH assay conditions were described above.

### Effect of Inducers

Various phenolics and non-phenolic inducers such as, 2,5 Xylidine, ferulic acid, p-cresol, p-anisidine, pyrogallol, 2-HBT, guaiacol, and ethanol were incorporated in GY medium at 1 mM final concentration along with 500 μM CuSO_4_ on 3rd day culture of MSF2. Ferulic acid, p-cresol, p-anisidine, 2-HBT, and guaiacol were dissolved in 50% ethanol whereas pyrogallol was dissolved in water. All the inducers were filter sterilized and used. To find out the optimum concentration of the inducer, 2,5 Xylidine was added at concentrations from 0.2 to 2 mM, on 3rd day of inoculation of MSF2 in GY medium and incubated under the conditions previously described.

### Substrate Specificity

In order to evaluate the substrate specificity of LccH, activities were measured using substrates such as cinnamic acid derivatives (ferulic acid, tannic acid, and vanillic acid), monophenols (p-cresol, 2-6 di methoxy phenol), o methoxy phenol (guaiacol, L-tyrosine), Diphenols (L-DOPA, Catechol), triphenol (pyrogallol) at 3 mM concentration under standard assay conditions. Relative activity was calculated for each substrate considering the activity on ABTS as 100%.

### Characterization of LccH

UV-Visible spectrum of ion-exchange purified LccH from *H. hirta* was analyzed in Cary 60 spectrophotometer from Agilent technologies USA. The molecular mass of LccH was determined in denaturing 12% sodium dodecyl sulphate-polyacrylamide gel electrophoresis (SDS-PAGE) (Laemmli, 1970). After electrophoresis, the gel was stained with Coomassie brilliant blue R-250 for visualization of total protein. The zymogram of native PAGE and visualization of in-gel LccH activity was carried out by incubating the gel in 50 mM citrate phosphate buffer (pH 3.4) containing 5 mM ABTS at 30 °C and same was later stained with Coomassie brilliant blue for total protein.

### Sequence and Structure Analysis

The computer program ‘Modeler’ was used for comparative protein structure modeling (Sali and Blundell, 1993; Fiser et al., 2000) in which the input was an alignment of a sequence to be modeled with the template structures of known atomic coordinates. The protein sequence of laccase-1 of the *Agaricus bisporous* (gi|568441732) was retrieved from NCBI. A search for potentially related sequences of known structure was performed by aligning the target sequence against the PDB database using p-BLAST and a potential template structure was identified as PDB:3KW7_A from *Trametes sp*. AH28-2. Comparative model was constructed for laccase-1 of *Agaricus bisporous* using PDB: 3KW7_A as a suitable template.

### Delignification of Woody Biomass and Corncob

Biomass samples of wood (*Melia dubia*) and corn cob were milled to 200 μm particle size using Willey Mill. Crude LccH (50 U.g^-1^ of dry biomass) in citrate phosphate buffer (pH 3.4) was treated with biomass at 5% consistency for 17 h at 40°C under shaking conditions (150 rev/min) in an environmental shaker. After incubation the solids were separated by centrifugation at 10,000*g* for 10 min and dried at 45°C in the vacuum oven for constant weight. Dried biomass (300 mg) was used for estimation of klason lignin (Rico et al., 2014). Proximate analysis of the biomass was carried out by following the procedure of National Renewable Energy Laboratory (NREL) (Sluiter et al., 2010).

## Results

### Screening and Molecular Identification of Isolate MSF2

**FIGURE 1:**
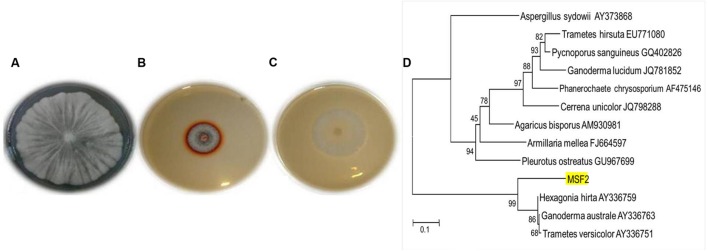
**Morphology, plate screening and identification of MSF2 **(A)** morphology of fungal isolate MSF2, **(B)** guaiacol oxidation in PDA medium and **(C)** PDA medium without guaiacol.** Reddish brown colouration due to oxidation by MSF2 in medium containing guaiacol under culture conditions at 30°C. **(D)** Phylogenetic relationship of isolate MSF2 and related species based on 18 S rRNA sequences. The numbers are accession numbers of 18 S rRNA sequences. Bootstrap values at nodes are percentage of 1,000 replicates. Scale bar indicates base substitutions/100 bases.

The WRB are the most widely studied efficient degraders of lignin by producing various lignin degrading enzymes; of which laccases play a vital role with multiple applications. The present investigation yielded an isolate MSF2, a non-spore forming hyaline fungi which showed highest guaiacol oxidation by forming a reddish brown zone around the colony in PDA plates (**Figures 1A–C**). Based on the sequence homology and identity, MSF2 showed 99% similarity to *Hexagonia hirta* (GenBank accession KT956905) followed by *Ganoderma australe* and *Trametes versicolor* (**Figure 1D**).

### Laccase Production by *H. hirta* MSF2

**FIGURE 2:**
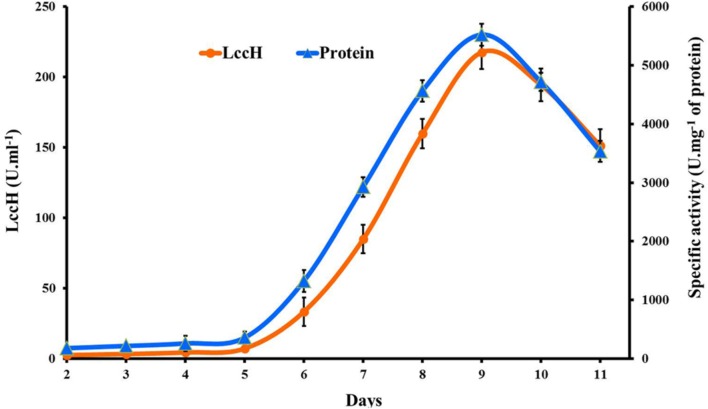
**Time course of volumetric and specific activity of laccase production by *H.hirta*.** Error bars represent standard deviations of triplicate experiments.

Laccase (LccH) production by *H. hirta* was monitored for 10 days in PDB medium. LccH activity was minimal (2.5 U.ml^-1^) on 2nd day and thereafter the activity gradually increased to maximum (217.59 U.ml^-1^) on 9th day and dropped subsequently (**Figure 2**). While comparing the specific activity of LccH, the trend appears to be similar but higher than volumetric activity which was 5522 U.mg^-1^ of protein on 9th day of cultivation.

### Standardization of Optimal Assay Conditions

**FIGURE 3:**
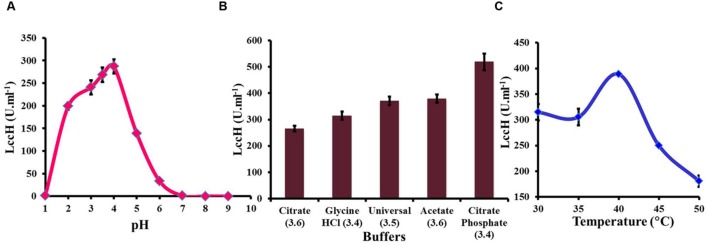
**Optimization of assay conditions for LccH activity **(A)** pH, **(B)** buffers, and **(C)** temperature.** **(A)** Effect of pH on LccH activity determined using universal buffer (pH range of 1–9). **(B)** Effect of different buffers such as citrate (pH 3.6), glycine-HCl (pH 3.4), universal (pH 3.5), acetate (pH 3.6), and citrate phosphate (pH 3.4) and **(C)** Effect of LccH activity at different temperature (30–50°C). Activity determination was carried out using 3 mM ABTS as substrate. Error bars indicate standard deviations of triplicate experiments.

Determination of LccH activity in the pH range of 1–10 exhibited a bell shaped profile with an optimum pH range of 3–4 (**Figure 3A**). The pH optima for LccH activity was 3.5 (268.52 U.ml^-1^). Increase in pH towards a neutral range resulted in a sharp decline in the activity and reached almost an undetectable level above pH 7.0. Different buffers tested in the pH range of 3 to 4 for optimal LccH activity showed that citrate phosphate buffer at pH 3.4 yielded highest (518.52 U.ml^-1^) activities (**Figure 3B**). To find out the optimum assay temperature for LccH activity, appropriately diluted enzyme in citrate phosphate buffer (pH 3.4) was assayed at different temperature ranges from 30 to 45°C. The results indicated a maximum (388.9 U.ml^-1^) LccH activity at 40°C (**Figure 3C**).

### Optimization of Media for LccH Production

**FIGURE 4:**
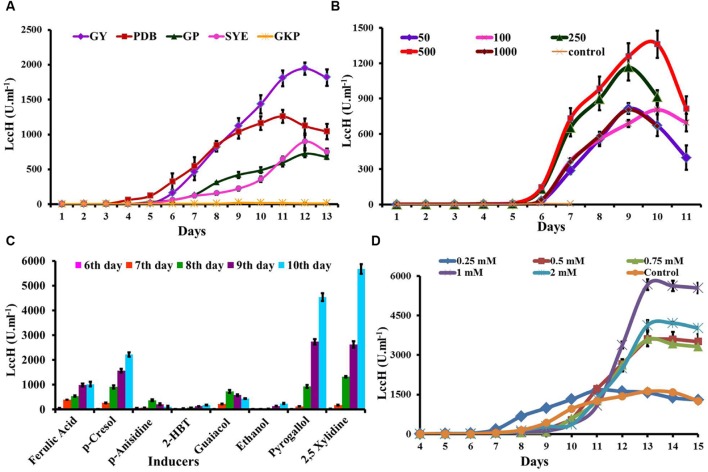
**Optimization of media and inducers for LccH production.** **(A)** The *H. hirta* MSF2 was grown in different media *viz*., PDB, GY, SYE, GKP, and GP at 30°C under static conditions and culture supernatants were assayed for LccH activity at standard assay conditions. **(B)** Optimization of CuSO4 concentrations for LccH production on GY medium. **(C)** Effect of inducers on LccH activity. All the inducers were evaluated at 1 mM concentration along with 500 μm of CuSO4. **(D)** Effect of different concentration of 2,5 Xylidine on LccH activity. Error bars represent standard deviations of triplicate experiments.

Five media were evaluated for LccH production by *H. hirta* MSF2. The fungus was able to grow well and secrete extracellular LccH at considerable quantities in all the media, except GKP (**Figure 4A**). Maximum LccH activity (1944.44 U.ml^-1^) was observed with GY medium followed by PDB (1259.26 U.ml^-1^). The LccH synthesis in GY medium initiated on 4th day and increased from 5th day (8.8 U.ml^-1^) and reached a maximum on 12th day and declined thereafter. In addition, appreciable LccH activity was also noticed in SYE (898.15 U.ml^-1^) and GP (724.54 U.ml^-1^) on 12th day, while minimal LccH activity was observed in the GKP medium (22.2 U.ml^-1^) on 9th day (**Figure 4A**).

### Optimization of Copper Concentrations

LccH induction by CuSO_4_ was performed in GY medium with concentrations ranging from 50 to 1000 μM on 3rd day of inoculation. In general, Cu^2^ strongly stimulated the LccH production and in particular, CuSO_4_ at 500 μM induced LccH synthesis on 5th day of inoculation and reached maximum titre (1362 U.ml^-1^) on 10th day with 160-fold higher activity compared to control (8.33 U.ml^-1^) (**Figure 4B**). While higher Cu^2+^ concentrations did not significantly increase the LccH production.

### Effect of inducers

*Hexagonia hirta* produced higher titres of laccase without addition of aromatic inducers. However, addition of aromatic/phenolic compounds in the growth media along with CuSO_4_ showed enhanced LccH activity. It was interesting to note that 2,5 Xylidine incorporation recorded highest LccH activity of 5671 U.ml^-1^ on 10th day which was followed by pyrogallol (4537 U.ml^-1^). However, other inducers showed considerable effect on LccH induction were p-Cresol (2129 U.ml^-1^), ferulic acid (987 U.ml^-1^), guaicaol (435 U.ml^-1^), p-anisidine (370 U.ml^-1^), ethanol (240 U.ml^-1^), and 2-HBT (189 U.ml^-1^) (**Figure 4C**). As 2,5 Xylidine induced maximum laccase activity, hence, various concentrations from 0.2 to 2 mM was tested and the results revealed that 1mM recorded maximal LccH activity (**Figure 4D**).

### Physiological Behavior of *H. hirta* on GY Medium

**FIGURE 5:**
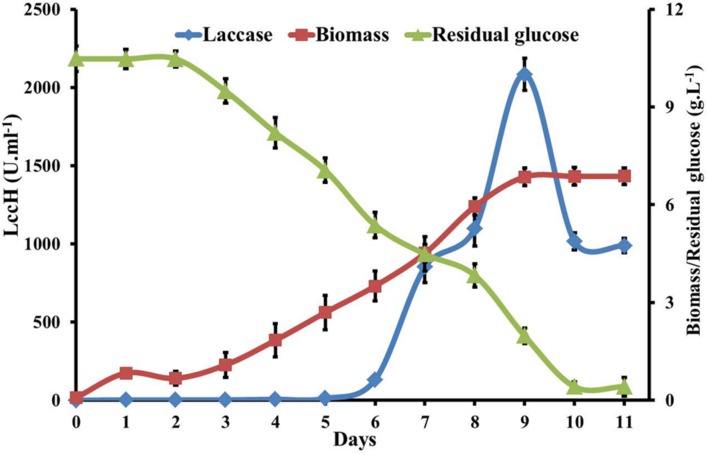
**Kinetics of glucose utilization, biomass and laccase production by *H. hirta.*** Initial substrate concentration 10.5g.L^-1^ culture MSF2 was incubated in GY medium with optimal CuSO4 and 2,5 Xylidine. Error bars indicate standard deviations of triplicate experiments.

Biomass production, laccase activity, glucose utilization, change in pH, and protein concentration in GY medium by *H. hirta* was evaluated for 12 days. LccH activity was initiated from 5th day (10.94 U.ml^-1^) of inoculation with biomass yield of 2.69 g.L^-1^ and reached a sharp increase of 2083 U.ml^-1^ on 9th day with a biomass yield of 6.85 g.L^-1^. Increased laccase production might be attributed to increased uptake of glucose by the fungus from 5th day onwards as evidenced by a twofold increase (2.75 to 5.13 g.L^-1^) in glucose consumption. Akin to laccase production, sugar consumption also followed a similar trend with a maximum of 8.5 g.L^-1^ on 9th day. Notably, even after a decline in LccH production (1016 U.ml^-1^) on 9th day, the biomass yield increased until 11th day (6.87 g.L^-1^) to complete glucose consumption (**Figure 5**). Nevertheless, the total protein concentration was very low for the entire period with a maximum of 0.4 mg.ml^-1^. With respect to pH, it dropped to 3.66 from an initial pH of 6.0.

### Substrate Specificity

**FIGURE 6:**
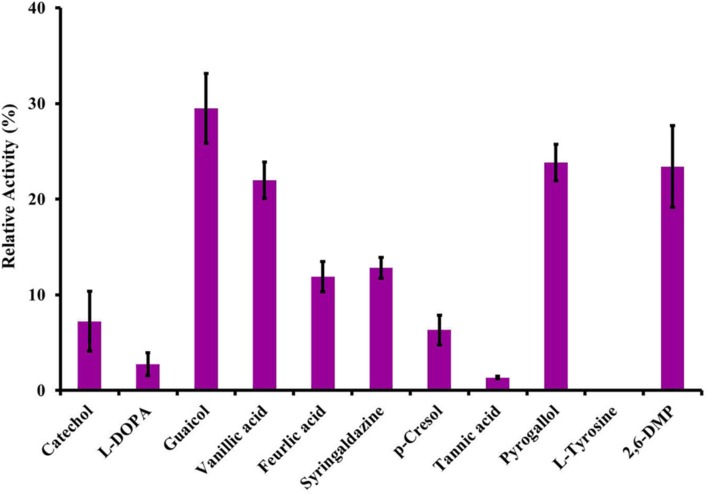
**Substrate specificity of LccH.** All the substrates were tested at 3 mM concentrations using citrate phosphate buffer pH 3.4 and the laccase activity was monitored under standard assay conditions. Activity with ABTS was considered as 100%.

Among the substrates evaluated, LccH utilized lignin and its related compounds. However, maximum catalytic activity was noticed for monophenols (guaiacol) followed by triphenols (pyrogallol, 2,6-DMP and vanillic acid) (**Figure 6**). Diphenols were poor substrates for LccH, lowering the catalytic efficiency by 2.73% for L-DOPA and 7.22% for catechol. Among the cinnamic acid derivatives tested for LccH activity, tannic acid was the least preferred to ferulic and vanillic acid. Also, LccH did not oxidize tyrosine (**Figure 6**) suggesting that the enzyme might belong to laccase class.

### Characterization of LccH

**FIGURE 7:**
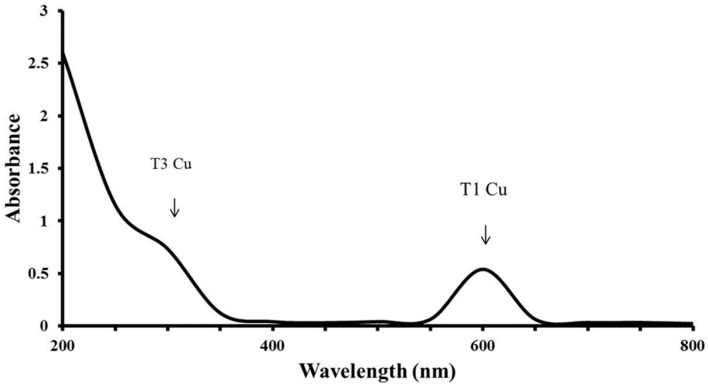
**UV-VIS absorbance spectrum of LccH.** A broad peak at 600 nm and a shoulder at 330 nm shows the T1 and T3 Cu^2^ center, respectively.

**FIGURE 8:**
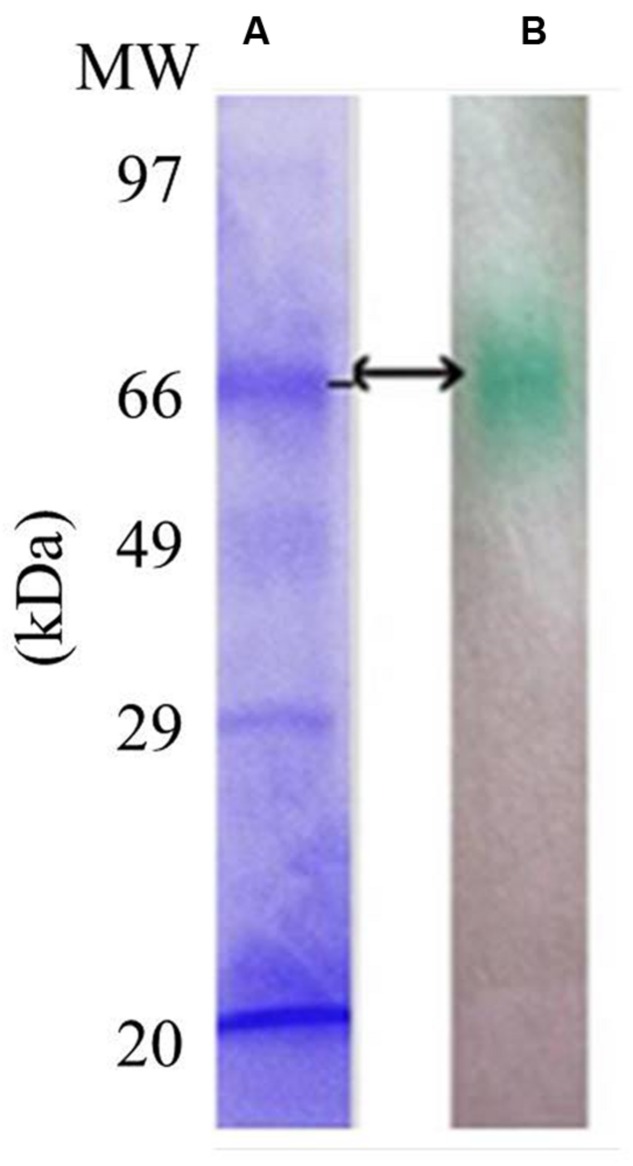
**Sodium dodecyl sulfate and Native PAGE of purified LccH.** For visualization of protein profiles **(A)** SDS gels were stained using coomassie brilliant blue R250 and **(B)** Zymogram of crude LccH with 5 mM ABTS as substrate.

LccH purified from *H. hirta* exhibited an absorbance spectrum typical of blue multicopper oxidases with a peak at 600 nm and a shoulder at 330 nm (**Figure 7**). Concentrated LccH fractions of MSF2 analyzed in SDS-PAGE showed a molecular mass of ∼66 kDa polypeptide and was confirmed further on Native PAGE by zymogram analysis using ABTS as substrate; the oxidized radical was visualized as a green colored band (**Figure 8**).

### Sequence and Structure Analysis

**FIGURE 9:**
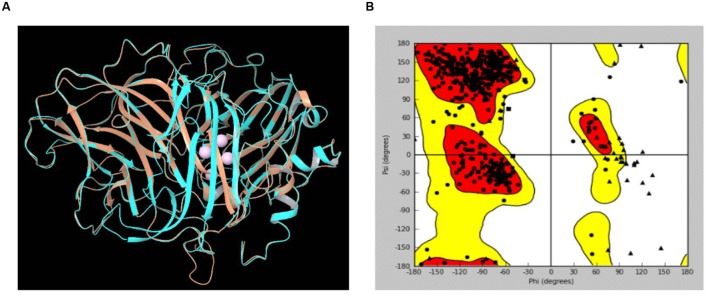
**(A)** Superimposed view of structural alignment between the laccase-1 model of *Agaricus bisporous* and PDB:3KW7_A [Cyan: laccase-1 model; Brown: 3KW7_A with four Cu ions in pink]; **(B)** Ramachandran Plot for laccase-1 model.

The LccH peptide mass fingerprinting matched with laccase-1 of *Agaricus bisporous* var. *bisporus* H97. The protein sequence of laccase-1 of *A. bisporous* (XP_006456378.1) shared an appropriate level of identity and similarity with *Trametes* sp. (PDB: 3KW7_A) (**Figure 9**). The reported Cu^2^ bound domain in the selected template PDB structure 3KW7_A chain were for Cu601:His400, Cys456 and His461; for Cu602:His64 and His403; for Cu603: His111, His405 and His455 and for Cu604: His66, His109 and His457 (Ge et al., 2010). The comparative protein structure model of laccase-1 of *A. bisporous* was built using Modeler with the template (3KW7_A) (Madhusudhan et al., 2006). The sequence identity between template and model was 48% (**Supplementary Figure S1**). The best structural model computed by modeler for laccase-1 showed a DOPE potential score of –49112.91 that signifies the satisfactory model. The superimposed view of structural alignment between the laccase-1 (gi| 568441732) model and PDB: 3KW7_A is shown in **Figure 9A** with a RMSD of 0.497Å. The Ramachandran plot showed more than 97% residues in the allowed/favorable region (**Figure 9B**).

### Delignification of Lignocellulosic Biomass

**FIGURE 10:**
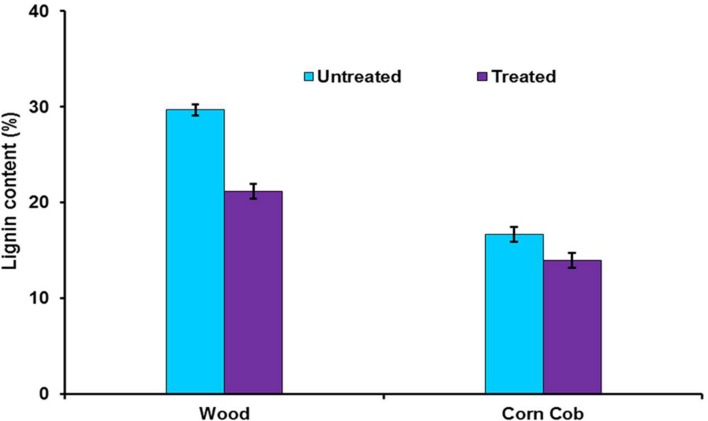
**Enzymatic delignification of wood and corncob using crude LccH.** The substrates were treated with crude LccH (50 μ.g^-1^ of biomass) for 17 h under shaking conditions.

To find out the effect of crude LccH on biomass pretreatment woody biomass and corn cob were separately treated with LccH. The initial lignin content of wood (29.7%) and corncob (16.7%) was reduced considerably achieving a delignification level of 28.6 and 16.5% for wood and corncob, respectively (**Figure 10**).

## Discussion

Laccases, being multifunctional and moonlighting biocatalysts with various commercial applications search for newer and novel laccase producing WRB for industrial applications is the current topic of interest to scientific community. Recently, many new laccase producing organisms have been reported (Kocyigit et al., 2012; Birhanli et al., 2013; Yang et al., 2013a; Dhakar and Pandey, 2015). In the present study, we report high level secretion of laccase by newly isolated *Hexagonia hirta* with a laccase (LccH) titre comparable and higher than the reports (**Table 1**). The higher laccase titres of *H. hirta* can be attributed to its source of isolation; it was isolated from an active wood degrading environment where, higher lignin content might have influenced *H. hirta* for its high level secretion.

**Table 1:** Literature comparison of laccase production by various WRB and recombinants.

Organism	Major components in the medium	Inducer	Laccase yield (U.ml^-1^) substrate used in the assay is given in parenthesis	Reference
*Trametes pubescens*	Glucose 40 g/L and Peptone 10 g/L	2 mM CuSO_4_	330 (ABTS)	Galhaup et al., 2002
		1 mM Gallic acid	350 (ABTS)	
		1 mM 2,5 Xylidine	275 (ABTS)	
*Pycnoporus cinnabarinus*	Maltose 20g/L	Ethanol 35 g/L	266 (ABTS)	Lomascolo et al., 2003
*Pleurotus ostreatus*	Glucose 10.0 g/L, peptone 0.5 g/L and 50 mg Vit. B1,	ABTS 1 mM,	400 (ABTS)	Hou et al., 2004
*Pycnoporus coccineus*	Glucose 10 g/L,	0.15 mM CuSO_4_ and 500 mM ethanol	100 (DMP)	Jaouani et al., 2005
*Tramets sp.* + *Trichoderma sp.*	Xylose 15 g/L and Tryptone 0.15%	1 mM CuSO_4_	184 (ABTS)	Zhang et al., 2006
*Cerrena unicolor*	Tomato juice medium	–	19 (ABTS)	Michniewicz et al., 2006
*Coriolopsis rigida*	Barley bran 50 g/L,	2 mM CuSO_4_ and 10 mM Xylidine	40 (ABTS)	Alcántara et al., 2007
*Ganoderma sp.*	Glycerol 40 g/L,	0.85 mM veratryl alcohol	240 (ABTS)	Teerapatsakul et al., 2007
*Lentinus strigosus*	Glucose 20 g/L,	2 mM CuSO_4_ and 2,6-dimethyl phenol	186 (ABTS)	Myasoedova et al., 2008
*S. ochraceum*	Glucose 20 g/L,	2 mM CuSO_4_ and 2,4-dimethyl phenol	33 (ABTS)	
WR1	Glucose	–	124 (ABTS)	Revankar and Lele, 2006
	Starch 2%	–	288 (ABTS)	
	Starch 2%	2 mM CuSO_4_	410 (ABTS)	
	Starch 2%	0.8 mM Xylidine	692 (ABTS)	
*Pleurotus ostreatus*	Glucose 10.5 g/L and Yeast extract 5 g/L	0.25 g CuSO_4_	150 (DMP)	Tlecuitl-Beristain et al., 2008
*Lentinus tigrinus*	Birch sawdust 20 g/L	1% butanol	24 (pyrocatechin)	Kadimaliev et al., 2008
*Cerrena unicolor*	Ethanol production residue (40 g/L),	0.5 mM TNT	165 (ABTS)	Elisashvili and Kachlishvili, 2009
*Cerrena* sp.	4 % PDB and 5 % soytone	0.4 M CuSO_4_ and 2 mM 2,5 Xylidine	202 (ABTS)	Chen et al., 2012
*Lentinus* sp.	2.4 % PDB and 5 % soytone	0.4 M CuSO_4_ and 2 mM 2,5 Xylidine	58 (ABTS)	Chen et al., 2012
*Shiraia* sp Super H-168	Starch 20 g/L and Yeast Extract 4 g/L,	0.6 mM CuSO_4_	101 (ABTS)	Yang et al., 2013a
**Recombinant laccases**				
*Schizophyllum commune* in *Aspergillus sojae*	Glucose 53 g/L, Tannic acid 25g/L	0.005 % CuSO_4_	770 (ADPB)	Hatamoto et al., 1999
*Trametes versicolor* in *Pichia pastoris*	Yeast nitrogen base with ammonium sulphate 13.4 g/L and biotin 400 μg/L	0.1 mM CuSO_4_ and 0.5 % Methanol	140 (ABTS)	Hong et al., 2002
*Trametes versicolor in Pichia methanolica*	Yeast Extract 10 g/L, peptone 20 g/L	0.2 mM CuSO_4_ and 0.8 % Methanol	12.6 (ABTS)	Guo et al., 2008
*Trametes sp.* 420 *in Pichia pastoris*	Methanol	0.3 mM CuSO_4_ and 0.6% alanines	83 (ABTS)	Hong et al., 2007
Recombinant *Haloferax volcanii*	Yeast-peptone-Casamino Acids	100 μM CuSO_4_	2.84(SGZ)	Maupin-Furlow et al., 2005
***Hexagonia hirta MSF2***	PDB	1 mM CuSO_4_	217 (ABTS)	**Present study**
	PDB	500 μM CuSO_4_	1259 (ABTS)	
	GY(Glucose 10.5 g/L and yeast extract 5 g/L)	500 μM CuSO_4_	1944 (ABTS)	
	GY(Glucose 10.5 g/L and yeast extract 5 g/L)	500 μM CuSO_4_ +1 mM 2,5 Xylidine	**5671.13 (ABTS)**	


*ABTS-2,2′-azino-bis-(3-ethylbenzothiazoline-6-sulfonic acid), SGZ-Syringaldizine, ADPB- 4-amino-2,6-dibromophenol, GY- Glucose Yeast Extract medium.*

Since the LccH was Cu^2^ dependent, Cu^2^ plays a key role as metal activator in the active site and induces both transcription and activity of laccase (Alvarez et al., 2009). In the present study, addition of Cu^2^ strongly stimulated the LccH production (**Figure 4B**), as observed in *Trametes versicolor* (Collins and Dobson, 1997), *Coriolus versicolor* MTCC 138 (Revankar and Lele, 2006) and *Phanerochaete chrysosporium* (Dittmer et al., 2009). In general, higher laccase activity was reported at Cu^2+^ concentrations ranging from 0.5 to 3.5 mM. Further, higher concentrations of Cu^2^ inhibits the growth and laccase production by generating hydroxyl radicals that directly damages DNA, proteins and membrane lipids (Revankar and Lele, 2006; Elisashvili and Kachlishvili, 2009; Bukh and Bjerrum, 2010; Elisashvili et al., 2010).

With the induction of Cu^2^, LccH production was attempted using various media, *H. hirta* MSF2 preferentially utilized simple carbon source such as glucose for maximal laccase yields. Most studies pertinent to higher laccase production used glucose as a carbon source (**Table 1**) than complex sources. Although *Cerena* sp WR1, preferred starch over glucose for laccase production (692 U.ml^-1^) (Revankar and Lele, 2006), much higher titres was obtained in the present study with *H. hirta* MSF2 on starch based media (898.15 U.ml^-1^). However, highest laccase activity (1944 U.ml^-1^) was recorded in glucose medium suggesting glucose as preferable substrate (**Table 1**; **Figure 4A**). The said LccH activity was obtained with the standardized assay conditions of pH 3.4 with citrate phosphate buffer at a temperature of 40°C. LccH was active at acidic pH as most fungal laccases function at acidic and near neutral pH, but lose their activities under alkaline conditions due to the reduction of ionization potential and the inhibition of -OH binding with T2/T3 Cu^2^ site (Zumarraga et al., 2008). LccH was optimally active at 40°C as reported for many laccases from *Rigidoporus lignosus* (Cambria et al., 2011) and *Cladosporium cladosporioides* (Aslam et al., 2012). The differences in laccase activity under different temperatures might be due to the number of disulphide bonds, thermal dissociation of the molecule and release of Cu ions at elevated temperature (Cruz Ramirez et al., 2012). Therefore, the property of thermo and solvent stability (unpublished results) of LccH makes it a potential candidate for most bioprocess and commercial applications.

Generally, WRB are known to secrete higher laccase yields ranging from 19 to 692 U.ml^-1^ under diverse nutritional and cultural conditions. Besides, various carbon and nitrogen sources at different concentrations, most of the studies presented in the table were under optimized conditions especially using many natural substrates (Birch saw dust, ethanol production residue, and barley bran etc.,) known for higher laccase production. The present study revealed that *H. hirta* MSF2 in GY medium supplemented with optimum Cu^2^ and 2,5 Xylidine produced a remarkable LccH activity (5671.13 U.ml^-1^), which is the first report to the best of authors knowledge.

UV Visible spectrum of LccH was similar to the reported fungal and bacterial laccase sharing four Cu^2^ atoms distributed in two centers namely type 1 and type 3 with all the conserved residues intact as evidenced by multiple alignments of protein sequences (**Supplementary Figure S1**). Many laccases isolated from fungi and bacteria are blue multicopper oxidases that coordinate four Cu^2^ atoms into three types of Cu^2^-binding sites (Bukh and Bjerrum, 2010). All of these ‘blue’ enzymes produce spectra with a maximum at 605 nm corresponding to the T1 Cu^2^ atom. The T2 Cu^2^ site exhibits weakly visible absorbance, and the T3 Cu^2^ site has two Cu^2^ centers and is responsible for a shoulder at around 330 nm. The spectrum of LccH is consistent with its clustering to blue laccases including the closely related and structurally characterized many fungal laccases (Baldrian, 2004; Chen et al., 2012). LccH produced was a monomeric protein as in many fungal laccases and their molecular mass ranged from 50 to 90 kDa (Baldrian, 2006). For instance, laccases from *Albatrella dispansus* (62 kDa) (Wang et al., 2004), *Coriolus zonatus* (60 kDa) (Koroljova et al., 1999), *H. echinaceum* (63 kDa) (Wang and Ng, 2004), *Marasmius quercophilus* (60 kDa) (Farnet et al., 2008), and *Trametes sanguinea* (62 kDa) (Hoshida et al., 2001) had a molecular mass very close to that of LccH. Further, mass spectrophotometric and comparative modeling results also confirmed monomeric state of LccH with RMSD of 0.497Å between the template and target model, which indicated their close structural alignment having all the 4 Cu^2^s conserved in a coordinated manner to exert the function.

Lignin removal from lignocellulosic feedstocks acts as a key challenge not only in bioethanol production but also releases sugars for further processing of cellulosic materials and recovers lignin for derived bioproducts and materials for various applications. Recalcitrance of biomass towards enzymatic hydrolysis mainly depends upon the lignin content and composition of the lignocellulosic materials (Studer et al., 2011). Enzymatic delignification is a substrate specific approach which effectively increases the reaction rate and delignification efficiency deprived of carbohydrate consumption (Vivekanand et al., 2008). Rico et al. (2014), studied delignification of *Eucalyptus* wood using recombinant *Myceliophtora thermophila* laccase at two doses 10 and 50 U.g^-1^ of dry biomass and achieved 22.1 and 28.4% of lignin removal, respectively. Similarly, delignification with elephant grass and eucalyptus using commercial laccase from *T. villosa* at 50 U.g^-1^ of dry biomass achieved 20.7% and 17.5% of reduction in lignin (Gutierrez et al., 2012), where as the present study used only crude LccH of 50 U.g^-1^ of biomass for delignification which is comparable with reported delignification rates. However, further optimization of the process using mediators would increase the lignin removal and recovery of lignin derived products. Nevertheless, these results depict the ability of LccH to remove lignin and generate of lignin derived aromatic chemicals.

## Conclusion

In this study, we report a high level laccase producing wood degrading basidiomycete, *H. hirta*, which produced 5671.13 U.ml^-1^ on 12th day in a GY medium supplemented with 500 μM CuSO_4_ and 1 mM 2,5 Xylidine. LccH is a monomer with molecular mass of ∼66 kDa, optimally active at 40°C, stable in many organic solvents and delignified the lignocellulosic substrates such as woody biomass and corncob, to a level of 28.6 and 16.5%, respectively. Considering high level secretion, stability and delignification ability, LccH from *H.hirta* could be a potential candidate for wide variety of biotechnological and industrial applications such as lignin derived products generations.

## Author Contributions

SK conducted study on isolation of fungi identification and laccase production. IM conducted experiments on laccase production, assay conditions, physiological behavior. SK and IM helped in drafting the manuscript. NP characterized the protein and assisted in experiments, AS conducted substrate specificity study and assisted in experiments, DS executed the bioinformatic works such as protein modeling and interpreted the data. SU conceived the idea, designed the experiments, wrote and reviewed the manuscript and coordinated the entire study. RK advised on the modification and inclusion of new experiments and reviwed the manuscript.

## Conflict of Interest Statement

The results of this study has been filed as an Indian patent in the name of Tamil Nadu Agricultural University, Coimbatore by the Inventors; SU, SK, IM, NP and RK, entitled ‘High level secretion and method of laccase production by *Hexagonia hirta* and uses there of ’ (645/CHE/2015). The remaining authors declare that the research was conducted in the absence of any commercial or financial relationships that could be construed as a potential conflict of interest.

## Footnotes

1 http://blast.ncbi.nlm.nih.gov/Blast.cgi

## Abbreviations

2,6-DMP-: 2,6 dimethoxy phenol
ABTS-2,2′: azino-bis-(3-ethylbenzothiazoline-6-sulfonic acid)
ADPB: 4-amino-2,6-dibromophenol
Cu^2+^: Copper
DNS: Dinitrosalicylic aicd
GKP: Glucose potassium peptone
GP: Glycerol Peptone
GY: glucose yeast extract
HBT: 2-Hydroxybenzothiozole
LccH: Laccase produced from *Hexagonia hirta;*
MSF2: Mettupalayam soil fungi 2
PDB: Potato dextrose broth
RMSD: Root means square deviation
SGZ: Syringaldizine
SYE: Starch yeast extract agar
WRB: White-rot basidiomycetes
