# Supplementary figures and images for: High Level Secretion of Laccase (LccH) from a Newly Isolated White-Rot Basidiomycete, Hexagonia hirta MSF2
Source: Front Microbiol. 2016 May 18;7:707. doi: 10.3389/fmicb.2016.00707 (PMC4870842; doi:10.3389/fmicb.2016.00707)

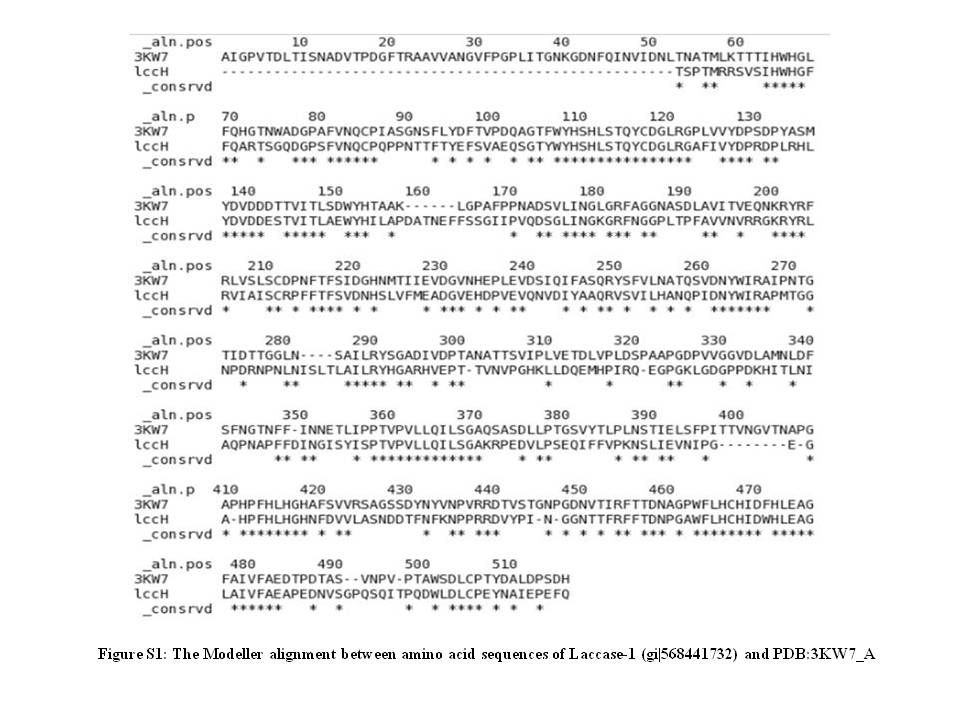

Supplement: Supplementary file 1 [file Image_1.JPEG]
